# Genomic and Transcriptomic Characterization of Canine Osteosarcoma Cell Lines: A Valuable Resource in Translational Medicine

**DOI:** 10.3389/fvets.2021.666838

**Published:** 2021-05-17

**Authors:** Cecilia Gola, Diana Giannuzzi, Andrea Rinaldi, Selina Iussich, Paola Modesto, Emanuela Morello, Paolo Buracco, Luca Aresu, Raffaella De Maria

**Affiliations:** ^1^Department of Veterinary Science, University of Turin, Turin, Italy; ^2^Department of Agronomy, Food, Natural Resources, Animals, and Environment, University of Padua, Padua, Italy; ^3^Faculty of Biomedical Sciences, Institute of Oncology Research, Universit‘a della Svizzera Italiana (USI), Bellinzona, Switzerland; ^4^National Reference Center for Veterinary and Comparative Oncology-Veterinary Medical Research Institute for Piemonte, Liguria, and Valle d'Aosta, Torino, Italy

**Keywords:** dog, osteosarcoma, cell line, next generation sequencing, whole-exome sequencing, RNA sequencing

## Abstract

Osteosarcoma (OSA) represents the most common primary bone tumor in dogs and is characterized by a highly aggressive behavior. Cell lines represent one of the most suitable and reproducible pre-clinical models, and therefore the knowledge of their molecular landscape is mandatory to investigate oncogenic mechanisms and drug response. The present study aims at determining variants, putative driver genes, and gene expression aberrations by integrating whole-exome and RNA sequencing. For this purpose, eight canine OSA cell lines and one matched pair of primary tumor and normal tissue were analyzed. Overall, cell lines revealed a mean tumor mutational burden of 9.6 mutations/Mb (range 3.9–16.8). Several known oncogenes and tumor suppressor genes, such as *ALK, MYC*, and *MET*, were prioritized as having a likely role in canine OSA. Mutations in eight genes, previously described as human OSA drivers and including *TP53, PTCH1, MED12*, and *PI3KCA*, were retrieved in our cell lines. When variants were cross-referenced with human OSA driver mutations, the E273K mutation of *TP53* was identified in the Wall cell line and tumor sample. The transcriptome profiling detected two possible p53 inactivation mechanisms in the Wall cell line on the one hand, and in D17 and D22 on the other. Moreover, *MET* overexpression, potentially leading to MAPK/ERK pathway activation, was observed in D17 and D22 cell lines. In conclusion, our data provide the molecular characterization of a large number of canine OSA cell lines, allowing future investigations on potential therapeutic targets and associated biomarkers. Notably, the Wall cell line represents a valuable model to empower prospective *in vitro* studies both in human and in dogs, since the *TP53* driver mutation was maintained during cell line establishment and was widely reported as a mutation hotspot in several human cancers.

## Introduction

Canine osteosarcoma (cOSA) represents the most common primary malignant bone tumor in dogs (1, 2) and is characterized by a natural history of disease and molecular abnormalities similar to human osteosarcoma (hOSA) (3, 4). cOSA is locally aggressive and highly metastatic (5), and despite significant improvements of surgical and chemotherapeutic treatments, most dogs perish within a year from the diagnosis (6), indicating a need for identification of specific tumor targets to develop novel treatment strategies. Recently, two whole-exome sequencing (WES) studies revealed that several pathways and driver genes, such as *TP53, RB1, DLG2, PTEN, MYC*, and *MET*, were equally mutated in both cOSA and its human counterpart (7, 8). Moreover, such genes have been previously identified as key players in cOSA pathogenesis (4, 9) and potential therapeutic targets (10–12). Two further studies characterized the genomic profile of several canine cancer cell lines, including cOSA cell lines, and investigated their relevance in comparative oncology (13, 14). Notably, driver mutations in MAPK/ERK and PI3K/AKT signaling pathways were identified in cOSA cell lines, and an anti-proliferative target inhibition using trametinib showed encouraging results, while alterations of the *TP53* pathway were detected in non-sensitive cell lines (13).

These data highlight the importance of canine cancer cell lines as effective and reproducible pre-clinical models to provide crucial insights on pathogenetic mechanisms and drug response (14). Even though canine cancer cells have been used in oncologic research over decades, their mutational profiles were never investigated thoroughly (15–17). Consequently, a deep mutational analysis of such *in vitro* models will allow the identification of new targets and offer valuable tools in translational medicine (18, 19), considering that integration of genomic data with drug screening is fundamental for the development and pre-clinical evaluation of novel treatments that would equally benefit canine and human patients.

The purpose of the current study was to describe the mutational landscape and determine variants and putative driver genes as well as gene expression aberrations by an integrated analysis of whole-exome and RNA sequencing of eight cOSA cell lines and one matched pair of primary OSA and normal tissue.

## Materials and Methods

### Sample Collection and Cell Culture

Eight primary canine OSA cell lines and one matched pair of FFPE primary OSA and normal tissue were analyzed.

Penny, Wall, Desmond, Sky, Lord, and Pedro cell lines were previously established and validated by Maniscalco et al., while D17 (ATCC® CCL-183™) and D22 (ATCC® CRL-6250™) were obtained from American Type Culture Collection.

These were cultured in Dulbecco's modified Eagle's medium (DMEM; D17 and D22) and Iscove's standard medium, supplemented with 10% fetal bovine serum (FBS), 1% glutamine, 100 μg/mL penicillin, and 100 μg/mL streptomycin. Cells were cultured at 37°C in a humidified atmosphere of 5% CO_2_. The FFPE samples were obtained from the same OSA from which the Wall cell line was established.

### DNA and RNA Isolation From Cell Lines and FFPE Tissues

Genomic DNA (gDNA) was isolated and purified from cell lines and the FFPE samples (Supplementary Table 1) using DNeasy Blood and Tissue kit (Qiagen, Hilden, Germany) and GeneRead DNA FFPE kit (Qiagen, Hilden, Germany), respectively. gDNA concentration was determined using the Qubit 2.0 Fluorometer (Thermo Fischer, Foster City, CA, USA). Total RNA was extracted from six cell lines (Penny, Wall, Desmond, Sky, D17, and D22; Supplementary Table 1) using QIAzol Lysis reagent (Qiagen, Hilden, Germany) and purified. The total RNA concentration was determined using the NanoDrop ND-1000 UV-Vis spectrophotometer, and its integrity was measured by the Bioanalyzer 2100 instrument (Agilent Technologies, Santa Clara, CA, USA). RNA samples with an RNA integrity number (RIN) ≥8 were considered for the RNA-seq library preparation.

The isolated DNA and RNA were stored at −20 and −80°C, respectively, until further use.

### WES and RNA-Seq Library Preparation and Sequencing

High-quality whole-genome libraries from 10 samples (eight cells lines and two FFPE samples) were prepared using the KAPA HyperPlus Library Preparation Kit (Roche Sequencing and Life Science, Wilmington, MA). Exome capture was executed using Roche's SeqCap EZ Share Prime Developer Kit (Roche Sequencing and Life Science, Wilmington, MA) for non-human genomes following the SeqCap EZ HyperCap Workflow User's Guide. Probes for the exome capture were designed on the target enrichment design of 150 megabases developed by Broeckx et al. (20). The developer's reagent (06684335001) was used in place of COT-1, and index-specific hybridization enhancing oligos were also used. The final concentration and size distribution were tested with the Bioanalyzer 2100 workstation (Agilent Technologies, Santa Clara, CA, USA). The libraries (fragments ranging from 300 to 400 bp) were then sequenced on an Illumina NovaSeq 6000 platform in a paired-end (150 PE) mode. The chosen target sequencing coverage was 200×. Non-normalized libraries for RNA sequencing experiments were prepared using NEBNext® Ultra™ II Directional RNA Library Prep (New England Biolabs) with Sample Purification Beads and NEBNext® Poly(A) mRNA Magnetic Isolation Module (New England Biolabs).

A single-end sequencing (75 SE) was carried out on a NextSeq 500 platform (Illumina Inc., San Diego, CA, USA).

### WES Data Analysis

Quality control prior to alignment was performed on output from Illumina software and was processed by FastQC v.0.11.9 (https://www.bioinformatics.babraham.ac.uk/projects/download.html) software. Trimmomatic was used to select high-quality reads and remove adapter sequences.

Filtered reads were mapped to the canine reference genome (CanFam3.1; Broad Institute, release 99) using BWA software (21). To verify coverage in the exonic regions, the resulting BAM files were manually inspected using Integrative Genomic Viewer (IGV) (22). Pre-processing for variant calling was performed following the Genome Analysis Toolkit (GATK) v.4.1 Best Practices (https://gatk.broadinstitute.org/hc/en-us/articles/360035894731-Somatic-short-variant-discovery-SNVs-Indels). Briefly, single-nucleotide variants (SNVs) and small insertion and deletions (indels) were identified with the GATK Mutect2 tool (23) and filtered for standard parameters of a min-alternate-count of 2, a min-alternate-frequency of 0.05, and a read depth > 10. To reduce germline artifacts, a panel of Normals (PON) was built using the GATK CreateSomaticPanelOfNormals tool by downloading public available WES data from 18 non-tumor-bearing and unrelated dogs (normal stroma and blood samples) from the NCBI SRA database (Supplementary Data 1) (13, 24). An additional filter was added to exclude known single-nucleotide polymorphisms as annotated in the dbSNP 146 using the Dog Genome SNP Database (http://dogsd.big.ac.cn/) (25). Distribution and functional consequences of variants were assessed using ANNOVAR. Additionally, missense mutations were categorized according to their pathogenicity using FidoSNP (26).

The detailed WES workflow applied to both canine OSA cell lines and the FPPE samples is summarized in Supplementary Figure 1.

### RNA-Seq Data Analysis

All RNA-seq analyses were performed using conventional RNA-seq analysis tools (27). Detailed information is provided in Supplementary Figure 2. Briefly, post-alignment quality parameters of RNA-seq (insert length, gene-mapping bias, RNA junctions) were evaluated using RSeQC (28) in standard mode. Next, the counts of aligned reads per gene were obtained using htseq-count from the HTSeq (29) software package in single-stranded mode. Only reads that were uniquely aligned were retained. Finally, count filtering and normalization were performed using EdgeR R package (30).

### Recurrent Variants and Putative Driver Mutations Identification

**Figure 1:**
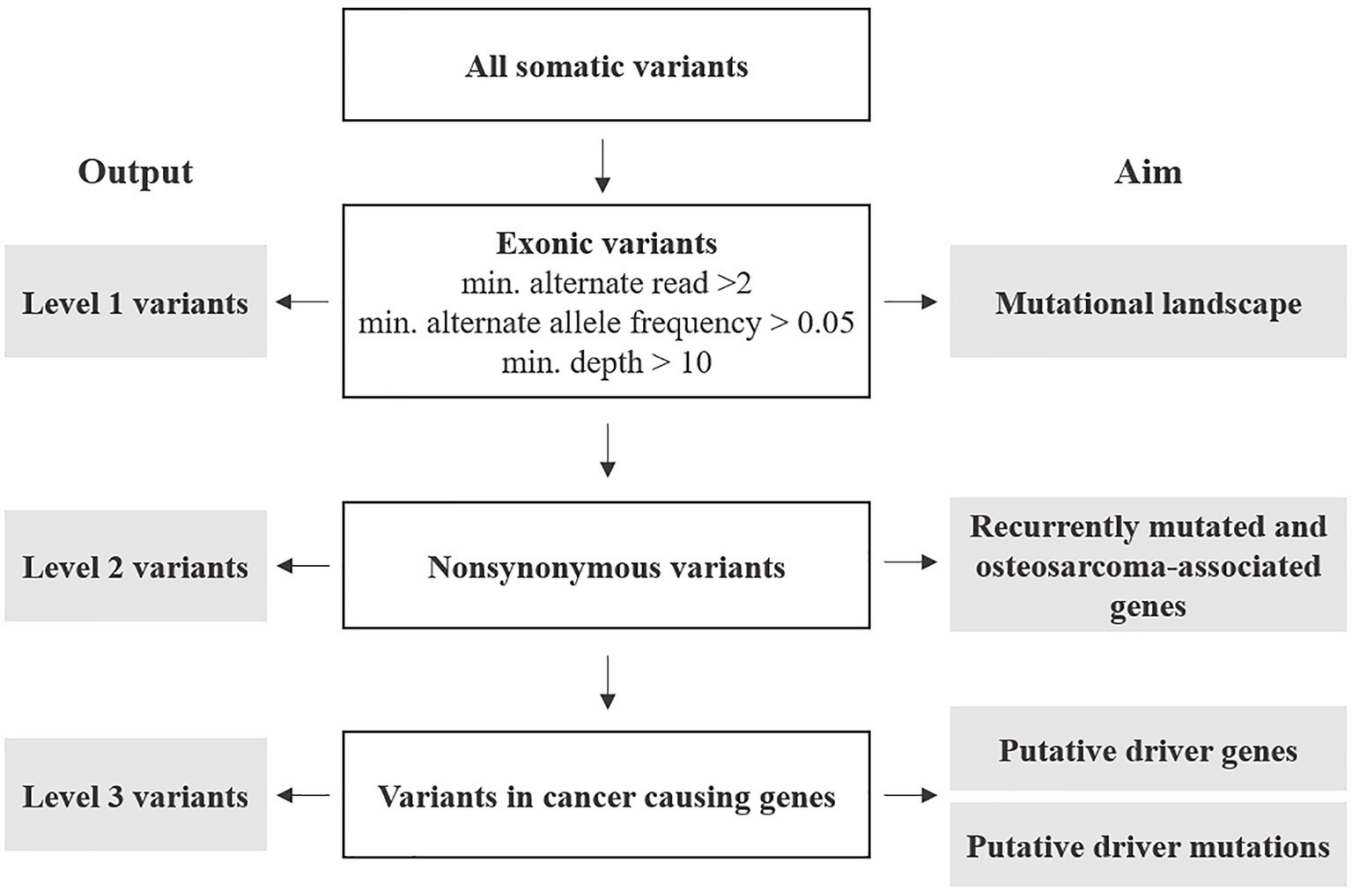
Post-processing of somatic variants: outline of selection criteria, categorization, and analyses.

Annotated variants were subjected to three filtering levels with increasing stringency and designated as follows (Figure 1 and Supplementary Data 2).

Level 1: variants included the totality of exonic-only SNV and indels retrieved from the variant call described above. These were further filtered for number of reads (min. 2), alternate allele frequency (min. 0.05), and each variant's depth of coverage (min. 10). The resulting variants were analyzed to describe the mutational profile of cOSA cell lines.

Level 2: these were non-synonymous exonic variants selected from Level 1 to identify recurrently mutated genes having a likely role in cOSA pathogenesis. Furthermore, variants encoding for genes commonly mutated in human and canine OSA were also prioritized (Supplementary Data 3) (7). Level 3: these were selected from Level 2 protein-coding variants of genes listed in COSMIC Cancer Gene Census, (version 92, https://cancer.sanger.ac.uk/census) (31). 5′ UTR and splice site variants COSMIC-listed genes were also included in the analysis due to their potential impact on protein expression and function. These variants were also manually cross-checked against known oncogenic variants in hOSA available on the IntOgen platform (https://www.intogen.org/search?cancer=OS) (32) to identify putative driver mutations.

### Validation of TP53 Mutation

E273K mutation of *TP53* identified in the Wall cell line and FFPE tumor sample was validated by Sanger dideoxy sequencing on Wall samples gDNA and Penny cell line (negative control). Briefly, two primers (sense 3′-ATGAGGGTGGCTAGGAGTCA-5′) and (antisense 5′-CAGTGCTGGGAAAGAGAGGA-3′) spanning the mutated region were designed by PRIMER3 Express software, and PCR on gDNA was performed using HotStar Taq (Qiagen) at 58°C (annealing temperature) for 35 cycles. After evaluation of agarose gel, amplification products were purified by QIAquick PCR Purification Kit (Qiagen).

## Results

### The Mutational Profile of Primary Canine Osteosarcoma Cell Lines

DNA extracted from eight canine OSA cell lines underwent WES, and an average of 158 million reads per sample (range 143–164) was obtained.

The mean depth of reads mapping to the canine reference genome (CanFam3.1) was 187.7 (range 128–219), with over 99% of the targeted exome uniquely aligned. The optimal coverage was achieved for six out of eight cell lines. For each cell line, all reads passed the quality control (Phred quality score) ≥ 30. The FFPE samples (tumor and matched normal) achieved a mean depth of 54.71.

**Figure 2:**
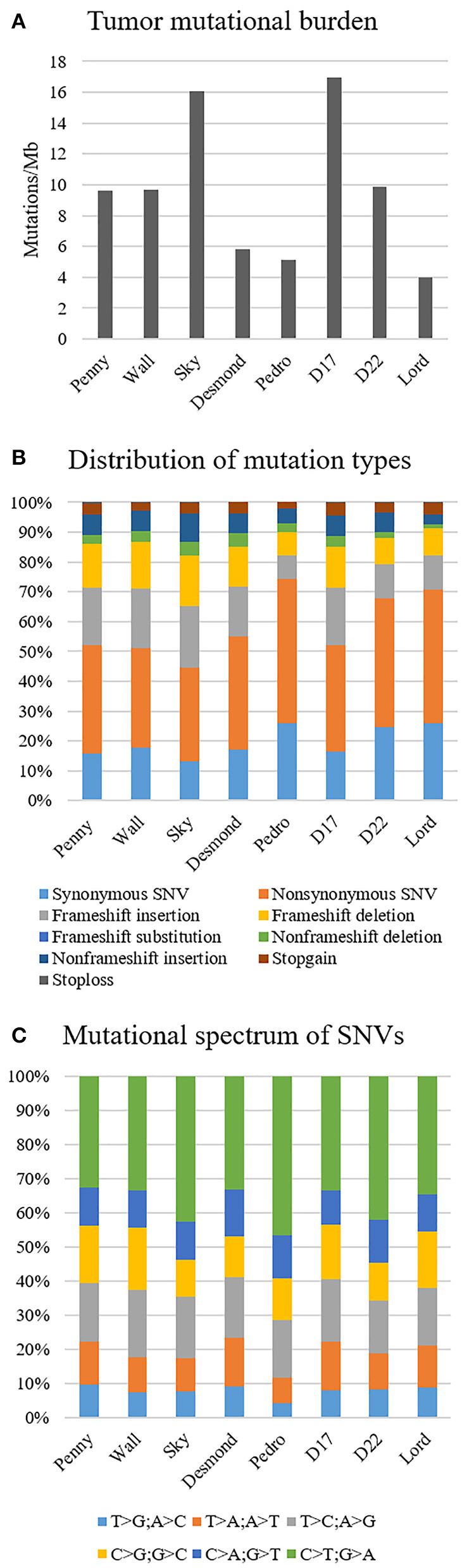
Mutational landscape of canine osteosarcoma cell lines, Level 1 variants. **(A)** Tumor mutational burden. **(B)** Distribution of mutation types. **(C)** Mutational spectrum of single-nucleotide variants.

The median tumor mutational burden of all Level 1 somatic variants was 9.6 mutations/Mb (range 3.9–16.9); in the Wall FFPE sample, the mutational burden reached 17.7 mutations/Mb (Figure 2A). On average, 19.6% (range 13.3–26%) and 17.1% of Level 1 variants of canine OSA cell line and Wall FFPE sample, respectively, were annotated as synonymous and were consequently excluded from downstream analyses.

In all our cell lines, missense mutations were the most frequently represented somatic coding mutation type, accounting for an average of 38.7% across all exonic variants. Frameshift insertion and deletions were 15.8 and 12.5% of the variants, respectively (Figure 2B). In Wall FFPE sample non-synonymous variants, frameshift and non-frameshift deletions were the most represented mutation types (36.3, 20.5, and 10.3%, respectively).

**Figure 3:**
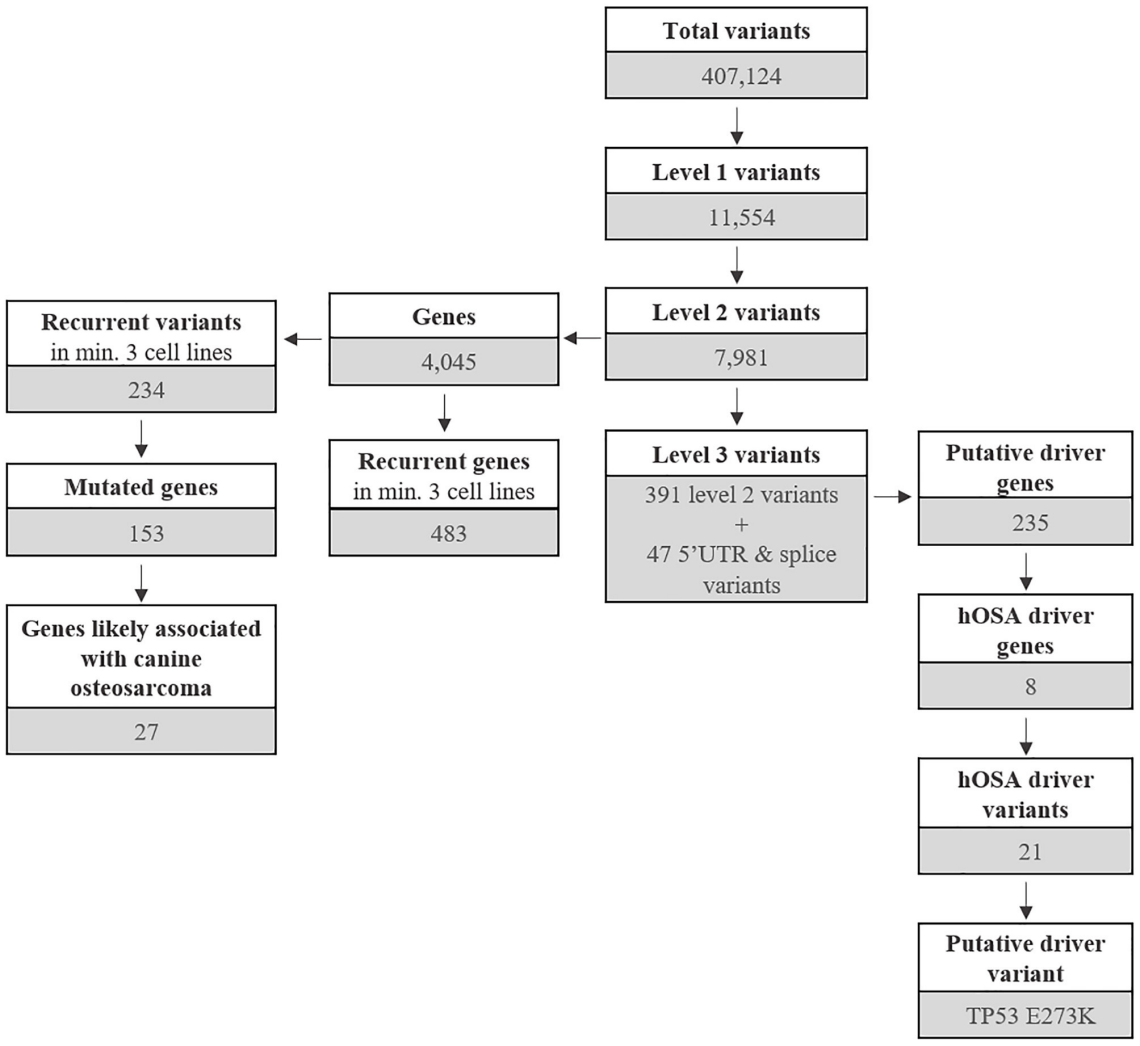
Outline of mutated genes and variants distributed across Level 1, 2, and 3 variants.

**Figure 4:**
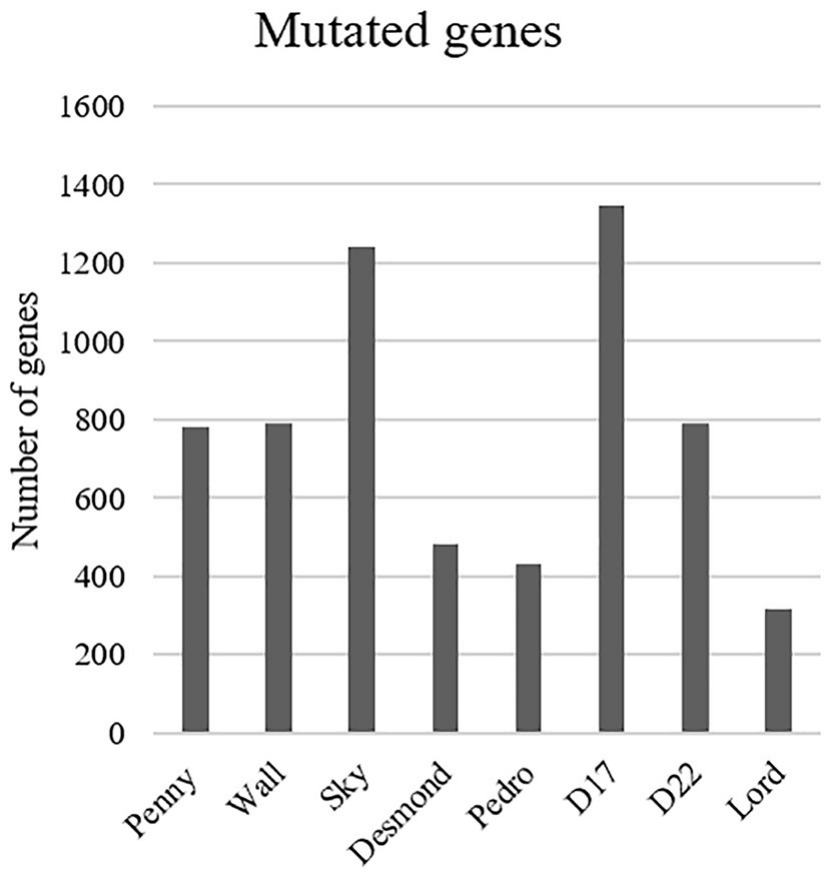
Distribution of the mutated genes across the cell lines.

The most common base change identified in all samples was C > T transition (Figure 2C). The analysis of the WES data using Mutect2 revealed a total of 11,554 exonic variants (Level 1); 7,981 of these were identified as non-synonymous (Level 2) and encoded for 4,045 genes (Figure 3). The number of genes in each sample ranged from 318 (Lord) to 1,345 (D17) and reached the maximum of 1,533 genes in the Wall FFPE sample (Figure 4).

Using the FidoSNP pathogenicity prediction tool, 50.5% (1,929/3,819 SNVs) of all missense point mutations were categorized as deleterious.

### Canine Osteosarcoma Cell Lines Show Mutations in Several Known Oncogenes and Tumor Suppressor Genes

**Figure 5:**
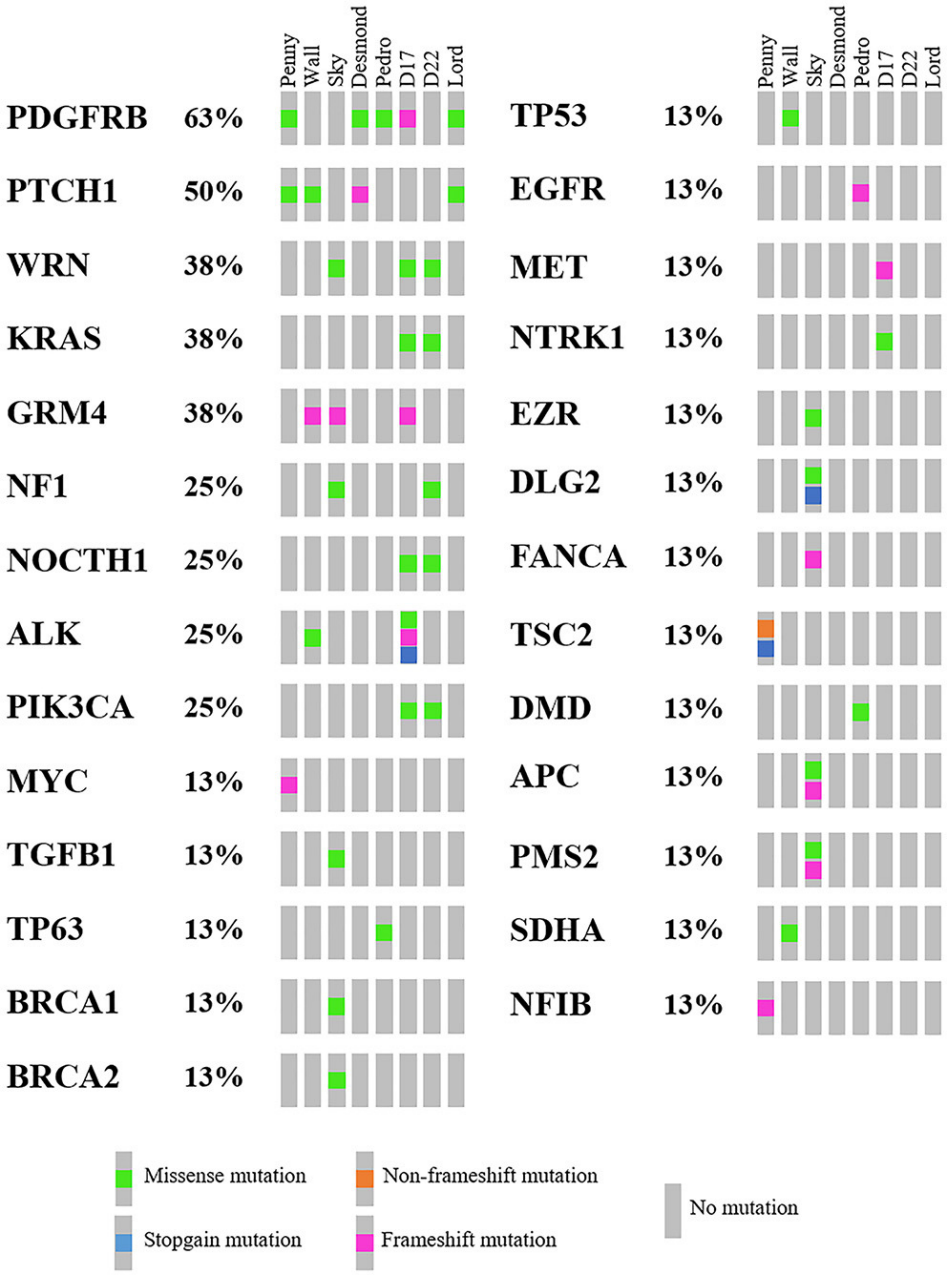
Oncoplot of genes likely involved in canine osteosarcoma pathogenesis retrieved in Level 2 analysis, including the mutational incidence and the mutational type across the cell line panel.

As mentioned above, 4,045 genes with 7,981 protein-modifying variants were identified (Supplementary Data 2). Overall, 483 genes were recurrently mutated in at least three cell lines. Taking into account the recurrent variants across all cell lines, a total of 234 variants were retrieved in three or more samples, and 51.4% of all SNVs (54/105) were categorized as pathogenic. When recurrent variants were collapsed to genes, 153 recurrently mutated genes were identified (Figure 3). In addition, genes were filtered using the list of osteosarcoma-associated genes retrieved from literature. Finally, a total of 27 genes likely implicated in OSA pathogenesis were identified across all our cell lines. Among them, *PDGFRB, PTCH1*, and *WRN* were retrieved in at least three cell lines, whereas oncogenes and tumor suppressor genes, such as *TP53, ALK, MYC*, and *MET*, were retrieved in only one cell line each (Figure 5).

The number of genes ranged from two (Desmond and Lord) to 10 genes (Sky) (Supplementary Figure 3). Overall, these cancer genes were encoded by 51 variants, and 53.3% of all SNVs (16/30) were categorized as pathogenic.

Comparing these 27 genes to the top 20 most frequently mutated genes in human cancers (Cancer Genome Atlas; https://portal.gdc.cancer.gov/), four overlapping genes were identified, namely, *PIK3CA, KRAS, APC*, and *NF1*, which ranked 2nd, 6th, 10th, and 13th, respectively. At last, four genes were also identified in the Wall FFPE sample but did not overlap those of the corresponding cell line (Supplementary Table 2).

### Canine Osteosarcoma Cell Lines Share Several Driver Genes With Human Osteosarcoma

COSMIC Cancer Gene Census was used to identify candidate driver variants (Level 3) in known cancer-causing genes in humans.

Level 3 included 438 variants coding for 235 genes, regardless of their incidence across the cell line panel (Figure 3 and Supplementary Data 2). A total of 19 genes were uniquely encoded by 5′ UTR or splice variants.

**Figure 6:**
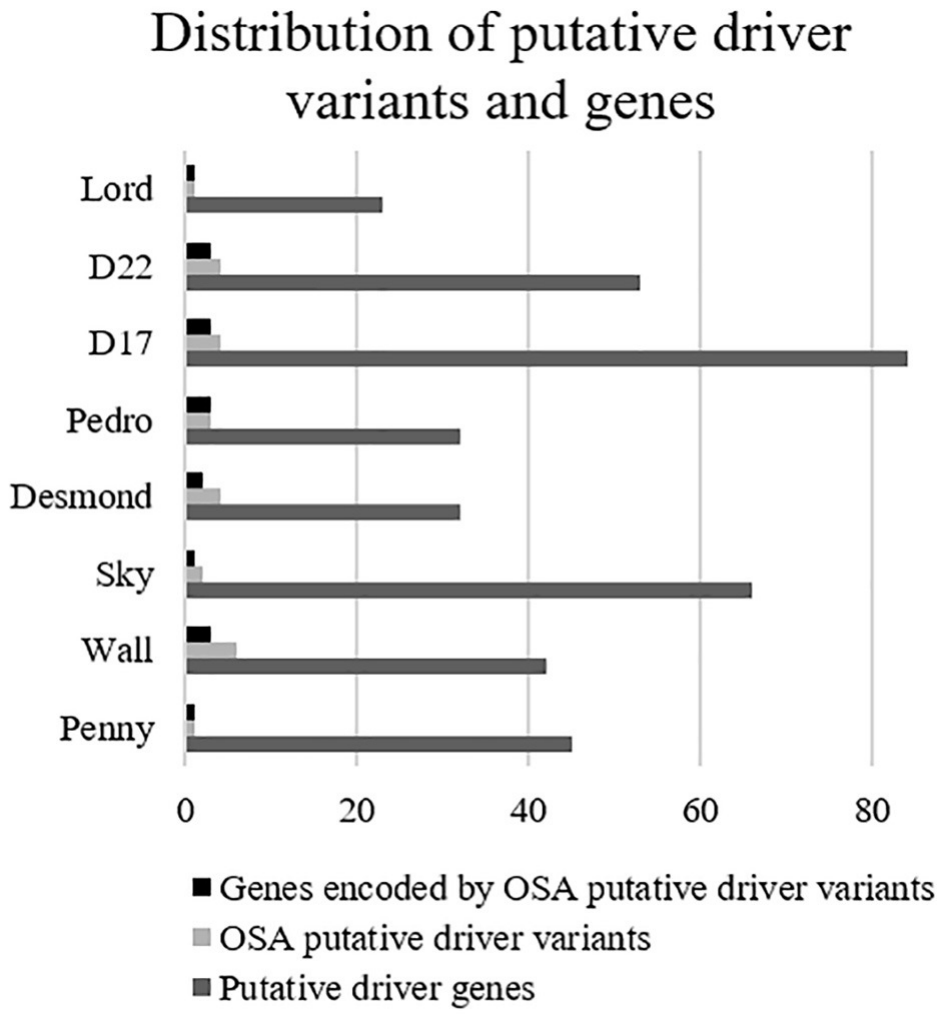
Distribution of putative driver variants and encoded genes across canine osteosarcoma cell lines.

Overall, 190 genes were indicated as having a relevant and documented activity that promotes oncogenic transformation. In particular, 28 were designated as fusion genes, 74 as tumor suppressors, and 63 as oncogenes, and 25 functioned as both. The distribution of putative driver mutations across all cell lines is depicted in Figure 6.

About 88% of the SNVs encoding for these genes were categorized as pathogenic.

When compared to the top 20 cancer driver genes involved in human OSA (COSMIC Cancer Browser), eight genes, encoded by 21 variants, were retrieved in Level 3 genes (Table 1). Among these, well-known oncogenes and tumor suppressor genes, such as *TP53, PTCH1, MED12*, and *PI3KCA*, were identified.

**Table 1:** List of putative driver genes and variants across all the cell lines.

**Gene**	**Mutation**	**Cell lines**							**FFPE**	
		**Penny**	**Wall**	**Sky**	**Desmond**	**Pedro**	**D17**	**D22**	**Lord**	**Wall**
PTCH1	c.17A>G	-	-	-	-	-	-	-	X	-
	c.3850C>T	X	-	-	-	-	-	-	-	-
	c.4014insT	-	X	-	-	-	-	-	-	X
	c.4023delA	-	X	-	-	-	-	-	-	X
	c.4200_4201insAGTCCCCG	-	X	-	X	-	-	-	-	X
	c.4203_4210del	-	X	-	X	-	-	-	-	X
LRP1B	c.12056A>T	-	-	-	X	-	-	-	-	-
	c.3112A>C	-	-	-	-	-	-	X	-	-
	c.3105_3106insATTGGGCCTGTGATGGTGA	-	-	-	-	-	-	X	-	-
ARID1A	c.6276A>T	-	X	-	-	-	-	-	-	X
	c.4863_4862insCCCCCCA	-	-	X	-	-	-	-	-	-
	c.4858_4852del	-	-	X	-	-	-	-	-	-
	c.1877G>A	-	-	-	-	X	-	-	-	-
NFATC2	c.510G>A	-	-	-	-	X	-	-	-	-
TET2	c.1349G>C	-	-	-	-	-	X	-	-	-
	c.2817_2818insCTGTGACTTCCTCCCTGGTCAGACA	-	-	-	-	-	X	-	-	-
	c.2894_2897del	-	-	-	-	X	-	-	-	-
PIK3CA	c.2217G>T	-	-	-	-	-	X	X	-	-
TP53	c.818C>T	-	X	-	-	-	-	-	-	X
MED12	c.2089_2090insATGGACTGCCCTTCCCCTCAC	-	-	-	X	-	-	-	-	-
	c.2581G>A	-	-	-	-	-	X	X	-	-

### Canine Osteosarcoma TP53 Putative Driver Mutation Matches a known Human-Equivalent Mutation Hotspot

All putative driver variants were cross-referenced with human OSA driver mutations available on IntOgen. Only the Wall cell line and FFPE tumor sample harbored a putative driver mutation, namely, the *TP53*^*E*273*K*^ mutation (c.818C>T) corresponding to the human *TP53*^E285K^ mutation hotspot.

**Figure 7:**
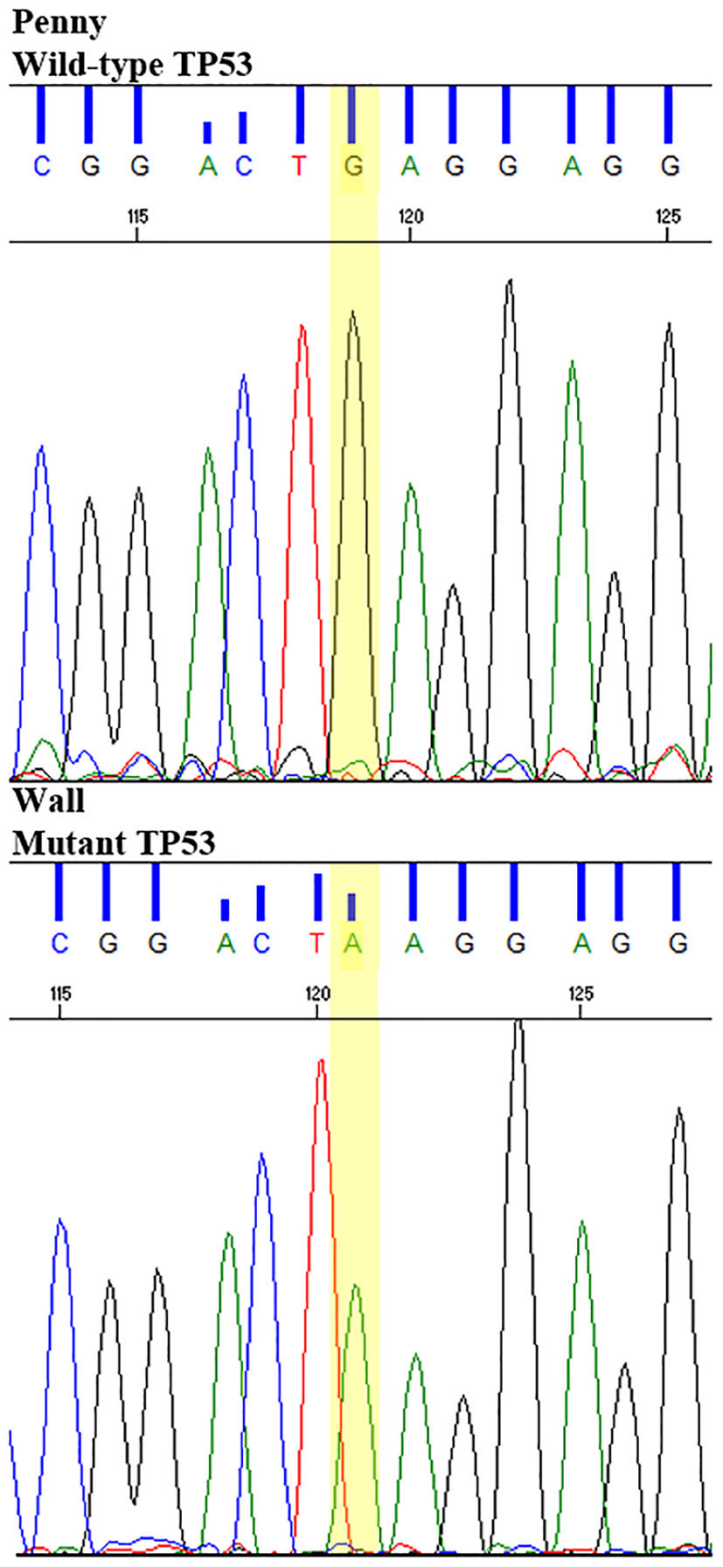
Sanger sequencing of TP53^E273K^ mutation in the Wall cell line and primary FFPE tumor, compared to wild-type TP53 Penny cell line and the reference canine TP53 sequence.

This putative driver mutation was further validated in homozygosis in the Wall cell line and tumor sample by Sanger sequencing (Figure 7).

### The Oncogenic Potential of TP53 and MET Gene Expression Aberrations

RNA sequencing generated over 10 million reads per sample. Quality control and trimming procedures retained the vast majority of the sequences, and unique alignment to the canine reference genome was successful for 86% of the cleaned reads (Supplementary Table 3).

The normalized gene expression of the osteosarcoma-associated genes described above was then profiled within the same gene across all samples.

In particular, *TP53* gene expression was increased in the Wall cell line, which harbored a hotspot mutation, as well as in D17 and D22, which retained a wild-type gene status. Interestingly, D17 and D22 showed a 9-fold and 5.5-fold increase of *MDM2* and *MDM4* transcript levels, respectively, compared to the other cell lines. Conversely, the *MDM2* transcript level was decreased by 2 to 20 times in the Wall cell line compared to the rest of the cell lines.

Transcriptional upregulation of *MET* by 19-fold was observed in the D17 cell line, which harbored a frameshift insertion on this gene, and to the same extent in D22 which retained a wild-type gene status. Notably, genes involved in the downstream MAPK/ERK pathway, such as *MAPK1, MEK*, and *MYC*, showed increased transcript levels, although no mutations were detected in WES analysis.

Conversely, downstream signaling components of mutated cancer-associated genes, such *as PTCH1, MED12, PDGFRB*, and *PIK3CA*, did not show any transcript level change.

## Discussion

Cancer cell lines are considered valuable models in basic cancer research, drug discovery, and translational medicine (14, 33, 34).

The recent characterization of a large panel of human cancer cell lines with omics technologies has empowered data-driven precision medicine (34, 35). Despite the substantial number of studies, an analogous dataset modeling canine cancer cell lines is currently unavailable (14).

In the present study, we integrated data from whole-exome and RNA sequencing of eight cOSA cell lines to obtain genomic and molecular data recapitulating *in vivo* cOSA pathogenesis and identifying suitable targets for drug discovery. To date, this dataset represents one of the largest explored for a single tumor in dogs.

Specifically, two commercial and six primary cOSA cell lines established and validated in our laboratory were analyzed. The assorted cell lines were previously used in several studies to investigate cOSA pathogenetic mechanisms and response to therapies (11, 12, 14, 36–38). So far, only the D17 cell line has been characterized at the genomic level by Das et al. in 2019 (13).

About exome sequencing, variants were analyzed using three levels of increased stringency: first, describing the mutational profile of each cell line; second, identifying recurrently mutated genes; and third, unraveling putative driver genes having a likely role in cOSA pathogenesis.

Overall, the mutational burden in our cOSA cell lines ranged between 3.9 and 16.9 mutations/Mb but was lower than the one described by Das et al. (13). However, the diversity of the cell lines and the differences in library preparation kits, exome capture designs, and downstream stringency filters may have caused this discrepancy.

Consistent with previous reports in cOSA cell lines and hOSA, the mutational type distribution showed a prevalence of missense mutations, and C>T transitions dominated this mutational spectrum (13, 39).

Across genes identified in Level 2 analysis, the two tyrosine kinase receptors *PDGFRB* and *MET* were retrieved. Both genes are known to play an important role in the development and progression of many canine cancers and were thoroughly investigated in cOSA as well (4, 11, 36, 40).

In five cell lines, *PDGFRB* harbored both frameshift and missense mutations, but only Desmond showed an increased gene transcript level. Nevertheless, no overexpression of *PDGFRB* downstream signaling molecules was detected, suggesting that these mutations did not affect gene transcription in Desmond. Gardner et al. reported previously that *PDGFRB* loci are affected by copy number gains rather than point mutations; however, no correlation with gene expression was found (7). The *MET* oncogene was highly expressed in D17 and D22 cell lines but resulted to be mutated only in the D17 cell line. However, the frameshift insertion mutation was unlikely associated with overexpression, since several stop codons were retrieved in the transcript analysis. Nevertheless, *MET* is regulated by several mechanisms, including amplifications and epigenetic aberrations (41). In D17 and D22 cell lines, overexpression of *MET* downstream genes, including *MAPK1* and *MEK*, was observed, suggesting a possible activation of the MAPK/ERK pathway (36, 42, 43).

The *MYC* gene was mutated in the Penny cell line only. However, increased transcript levels were identified in D17 and D22 cell lines and likely related to the aforementioned *MET* signaling. In hOSA cell lines, *MYC* overexpression promotes cell invasion *via* MAPK/ERK signaling and is correlated with a poor prognosis *in vivo* (44, 45). Such data are currently unavailable in cOSA, but aberrant activations of *MYC* and MAPK pathway genes have been reported (7, 13).

Mutations of *TP53* in Wall and *WRN* in the Sky, D17, and D22 cell lines were also retrieved. In particular, the putative driver *TP53*^*E*273*K*^ mutation was identified in the Wall cell line and tumor sample and further validated by Sanger sequencing. As most of the *TP53* mutations, *TP53*^*E*273*K*^ occurred in the mutational hotspot corresponding to the DNA-binding domain (46) and matched the human pathogenic hotspot mutation E285K (7). Both canine *TP53*^*E*273*K*^ and its human equivalent were previously reported in cOSA and hOSA (7). According to the IARC TP53 database (47), this mutant allele is listed among the top 15 most common mutations in human cancers predicted to disrupt protein structure and function (48). In our experiment, the presence of this mutation in both Wall tumor tissue and the derived cell line demonstrates a genetic fidelity with the primary tumor that remained stable during cell line establishment (34, 49).

Looking at gene expression, the mutant *TP53* transcript levels in Wall were twice as high as in Penny, Sky, and Desmond cell lines. This is in accordance with the literature where *TP53* missense mutations are reported to moderately affect the transcription but produce a full-length protein with a scarce ability to bind specific DNA sequence motifs and activate downstream target genes (48, 50). Concurrently, *MDM2*, a well-known *TP53* transcriptional target, showed a lower expression in Wall compared with the other wild-type *TP53* cell lines. This data suggests that *TP53* mutation in the Wall cell line might cause a loss of function rather than an altered mRNA expression (51). Overall, this indicates that *TP53*^*E*273*K*^ is a likely pathogenic driver mutation providing a spontaneously inactivated *TP53 in vitro* model for specific biological and reactivation assays (52).

Remarkably, D17 and D22 cell lines showed an increase of the *TP53* transcript compared to Wall, while retaining a wild-type gene status. In association, *MDM2* and *MDM4* transcript levels were increased, suggesting that *TP53* function might be impaired by their oncogenic and deregulated inhibiting activity in these cell lines (53). In accordance with our findings, *TP53* overexpression in D17 was also detected in a recent report by Modesto et al. (38).

Besides *TP53*, putative driver genes such as *PTCH1, MED12*, and *PIK3CA* were identified in Level 3 analysis. *PTCH1* was mutated in four out of eight cell lines. Physiologically, Hh ligand binding to the Ptch1 receptor relieves its inhibitory effect on the canonical Hedgehog pathway, whose activation plays a role in both hOSA and cOSA (10, 54). Despite a low *PTCH1* transcript level in these cell lines, no constitutive expression of Hedgehog pathway target genes was detected.

Similarly, *MED12* and *PIK3CA*, which are known to contribute to hOSA initiation and progression *via* the Wnt and PI3K/Akt pathways (55, 56), did not show gene expression aberrations in the mutated cell lines, suggesting a biological irrelevant role in our cell lines.

Contrary to previous reports in dogs, no somatic mutations neither gene expression aberrations affecting *CDKN2A* and *SETD2* were identified (7, 8). Regarding *CDKN2A*, it is generally affected by germline mutations and copy number variations, which were not investigated here (7, 8, 57), whereas *SETD2* mutations were only recently identified in hOSA and cOSA, and their biological role remains to be elucidated (7, 8, 58).

In conclusion, these data provide valuable insights into the molecular mechanisms of a large number of cOSA cell lines, allowing future investigations of their functional implications and drug response. Since similarities were identified with hOSA, these cell lines may also represent excellent translational models. In future, the addition of new primary cOSA cell lines and the integration of new sequencing approaches, such as methylation analysis and single-cell RNA-seq, are needed to provide an accurate characterization of these models and explore the underlying oncogenic mechanisms.

## Data Availability Statement

The datasets presented in this study can be found in online repositories. The names of the repository/repositories and accession number(s) can be found here: https://www.ncbi.nlm.nih.gov/, PRJNA701141.

## Author Contributions

RD and LA contributed conception and design of the study. PB and EM enrolled the canine osteosarcoma cases used for cell line establishment. CG, PM, SI, and RD cultured the cell lines and extracted DNA and RNA from canine osteosarcoma cell lines and FFPE samples. AR performed the NGS sequencing. DG performed the computational analyses. CG performed data post-processing. CG and SI performed data visualization. CG and DG wrote the manuscript. RD edited the manuscript. All authors contributed to the article and approved the submitted version.

## Conflict of Interest

The authors declare that the research was conducted in the absence of any commercial or financial relationships that could be construed as a potential conflict of interest.

## Supplementary Material

Supplementary Figure 1 Workflow for whole-exome sequencing short variant discovery, sequential use of tools in evaluation of canine osteosarcoma cell lines and FFPE DNA samples.

Supplementary Figure 2 Workflow for RNA sequencing analysis, sequential use of tools in evaluation of canine osteosarcoma cell lines.

Supplementary Figure 3 **(A)** Distribution of recurrently mutated genes and variants across all the canine osteosarcoma cell lines. **(B)** Distribution of osteosarcoma-associated genes and corresponding variants across all the canine osteosarcoma cell lines.

Supplementary Table 1 NGS sequencing technologies applied in the analysis of eight canine osteosarcoma cell lines and one matched FFPE tumor sample.

Supplementary Table 2 Variants retrieved in genes likely involved in canine osteosarcoma pathogenesis (*n* = 27; Level 2 analysis) in Wall cell line and matching FFPE tumor sample.

Supplementary Table 3 RNA sequencing quality control, trimming and alignment data.

Supplementary Data 1 Panel of Normals: public available WES data from 18 non-tumor bearing and unrelated dogs (normal stroma and blood samples) from NCBI SRA database.

Supplementary Data 2 Whole-exome sequencing results of 8 canine osteosarcoma cell lines and one matched FFPE tumor sample: Level 1, Level 2, and Level 3 variants.

Supplementary Data 3 List of known osteosarcoma-associated gene in dogs and humans.
