# Supplementary figures and images for: Genomic and Transcriptomic Characterization of Canine Osteosarcoma Cell Lines: A Valuable Resource in Translational Medicine
Source: Front Vet Sci. 2021 May 17;8:666838. doi: 10.3389/fvets.2021.666838 (PMC8165228; doi:10.3389/fvets.2021.666838)

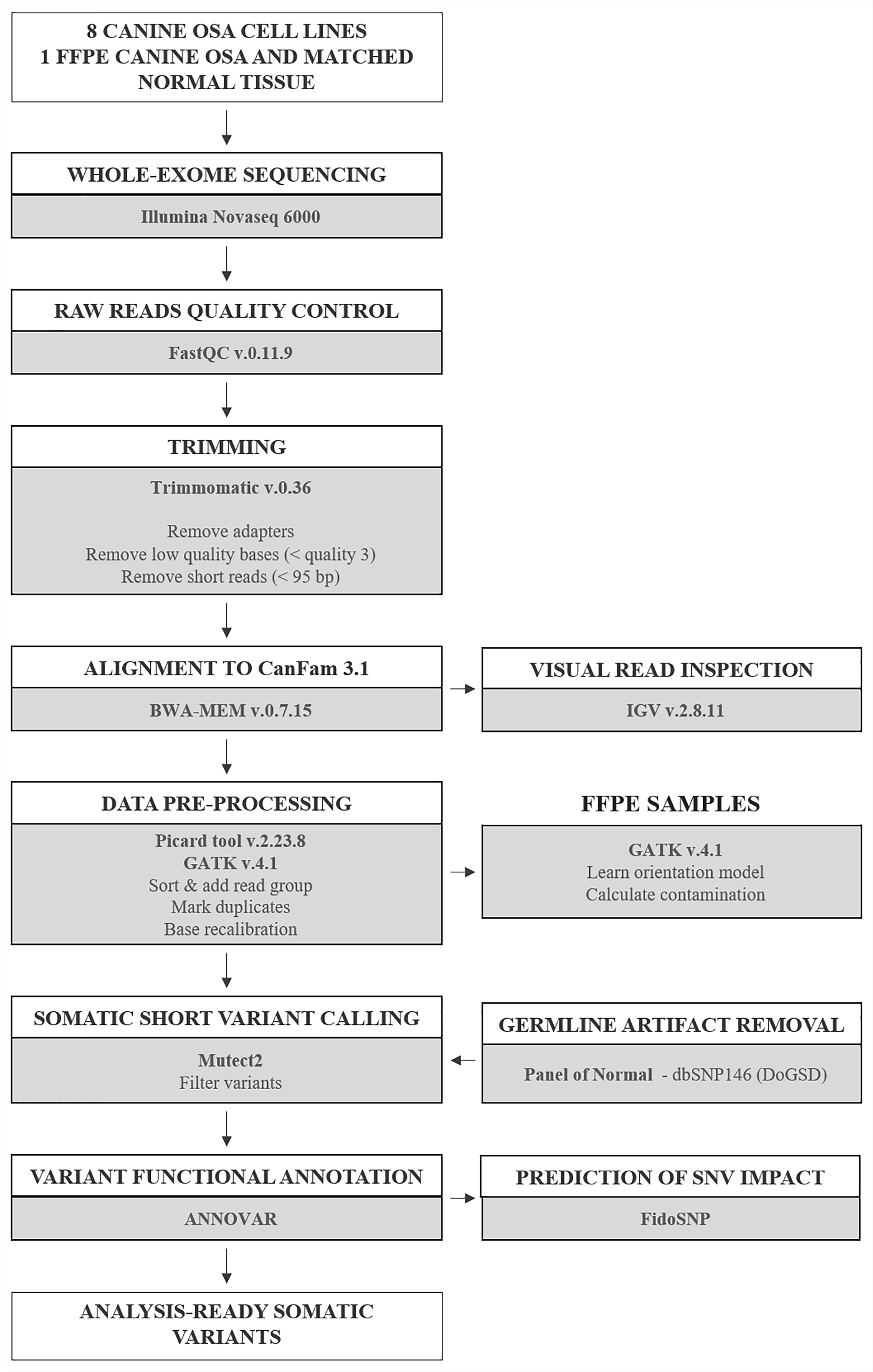

Supplement: Supplementary Figure 1 — Workflow for whole-exome sequencing short variant discovery, sequential use of tools in evaluation of canine osteosarcoma cell lines and FFPE DNA samples. [file Image_1.TIF]

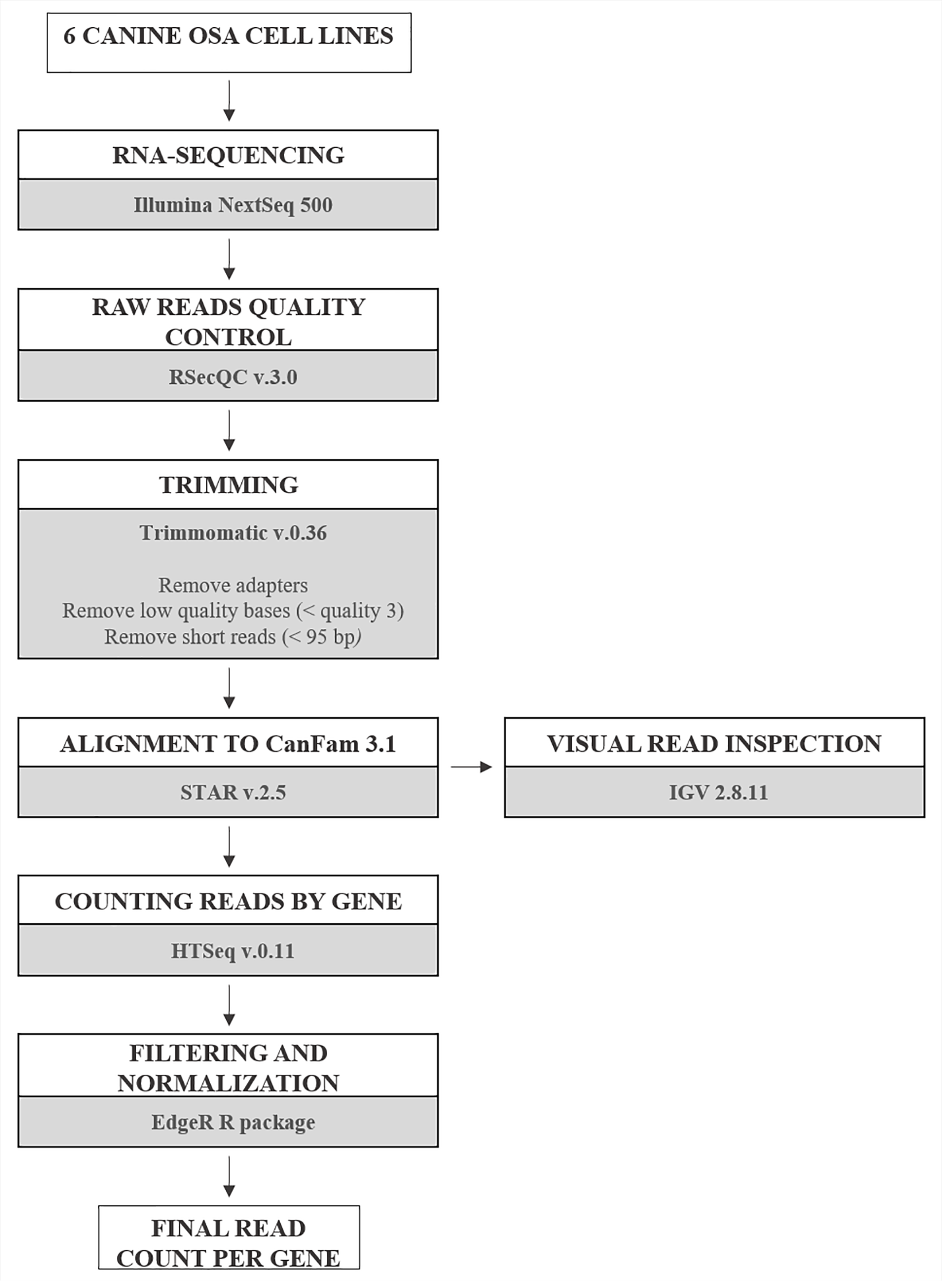

Supplement: Supplementary Figure 2 — Workflow for RNA sequencing analysis, sequential use of tools in evaluation of canine osteosarcoma cell lines. [file Image_2.TIF]

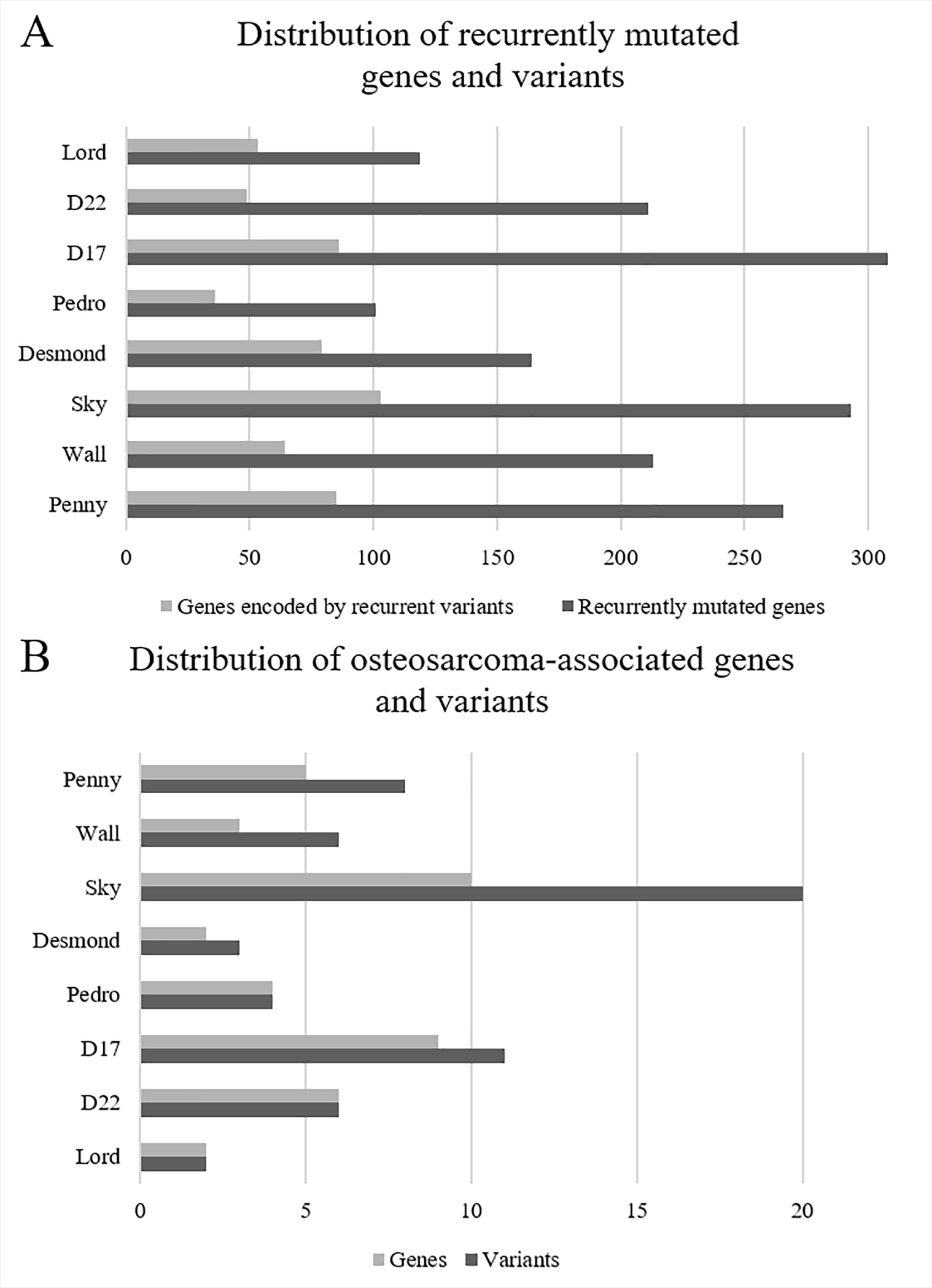

Supplement: Supplementary Figure 3 — (A) Distribution of recurrently mutated genes and variants across all the canine osteosarcoma cell lines. (B) Distribution of osteosarcoma-associated genes and corresponding variants across all the canine osteosarcoma cell lines. [file Image_3.TIF]
