# Supplementary material for: Genomic and Transcriptomic Characterization of Canine Osteosarcoma Cell Lines: A Valuable Resource in Translational Medicine
Source: Front Vet Sci. 2021 May 17;8:666838. doi: 10.3389/fvets.2021.666838 (PMC8165228; doi:10.3389/fvets.2021.666838)
Supplement: Supplementary Table 1 — NGS sequencing technologies applied in the analysis of eight canine osteosarcoma cell lines and one matched FFPE tumor sample. [file Data_Sheet_4.docx]

Supplementary Material

# Supplementary Tables

**Supplementary Table 1**NGS sequencing technologies applied in the analysis of eight canine osteosarcoma cell lines and one matched FFPE tumor sample.

|  | **Whole-exome sequencing** | | **RNA-sequencing** |
| --- | --- | --- | --- |
|  | Cell line | FFPE sample  (matched normal) | Cell line |
| **Penny** | X |  | X |
| **Wall** | X | X | X |
| **Sky** | X |  | X |
| **Desmond** | X |  | X |
| **Pedro** | X |  |  |
| **D17** | X |  | X |
| **D22** | X |  | X |
| **Lord** | X |  |  |

**Supplementary Table 2**Variants retrieved in genes likely involved in canine osteosarcoma pathogenesis (n= 27; Level 2 analysis) in Wall cell line and matching FFPE tumor sample.

| **Genes** | **Wall** | |
| --- | --- | --- |
|  | **Cell line** | **FFPE** |
| PDGFRB | - | - |
| PTCH1 | X | X |
| WRN | - | - |
| NOTCH1 | - | - |
| PIK3CA | - | - |
| NF1 | - | X |
| KRAS | - | - |
| ALK | X | - |
| GRM4 | - | - |
| TP53 | X | X |
| MYC | - | - |
| TGFB1 | - | - |
| TP63 | - | - |
| BRCA1 | - | - |
| BRCA2 | - | X |
| EGFR | - | - |
| MET | - | - |
| NTRK1 | - | - |
| EZR | - | X |
| DLG2 | - | - |
| FANCA | - | - |
| TSC2 | - | - |
| DMD | - | - |
| APC | - | - |
| PMS2 | - | X |
| SDHA | X | - |
| NFIB | - | - |

**Supplementary Table 3**RNA sequencing quality control, trimming and alignment data.

|  | **Raw reads** | **Trimmed reads** | **% of Raw** | **Aligned reads** | **Unique alignments** | **% on Raw** | **% on Aligned** |
| --- | --- | --- | --- | --- | --- | --- | --- |
| **Penny** | 9584204 | 9463443 | 98.74 | 9379884 | 8196928 | 85.53 | 87,38837 |
| **Wall** | 9914659 | 9914659 | 99.06 | 9753738 | 8593861 | 71.67 | 88,10838 |
| **Sky** | 11374245 | 10825893 | 95.18 | 10764666 | 9713074 | 85.40 | 90,23108 |
| **Desmond** | 9915411 | 9227281 | 93.06 | 9172818 | 8273072 | 83.44 | 90,19117 |
| **D17** | 11609741 | 91717 | 99.21 | 11400524 | 10207999 | 87.93 | 89,53974 |
| **D22** | 12488411 | 12357283 | 98.95 | 12228889 | 10938474 | 87.59 | 89,44781 |
